# 基于手性固定相的超高效液相色谱-串联质谱法检测小麦及其加工制品中的腈菌唑对映体

**DOI:** 10.3724/SP.J.1123.2021.03001

**Published:** 2021-07-08

**Authors:** Yanli QI, Jing GAO, Weirong WANG, Jing JIN, Ying LÜ, Shu QIN

**Affiliations:** 山西功能农产品检验检测中心, 山西农业大学, 山西 太原 030031; Shanxi Center for Testing of Functional Agro-Products, Shanxi Agricultural University, Taiyuan 030031, China; 山西功能农产品检验检测中心, 山西农业大学, 山西 太原 030031; Shanxi Center for Testing of Functional Agro-Products, Shanxi Agricultural University, Taiyuan 030031, China; 山西功能农产品检验检测中心, 山西农业大学, 山西 太原 030031; Shanxi Center for Testing of Functional Agro-Products, Shanxi Agricultural University, Taiyuan 030031, China; 山西功能农产品检验检测中心, 山西农业大学, 山西 太原 030031; Shanxi Center for Testing of Functional Agro-Products, Shanxi Agricultural University, Taiyuan 030031, China; 山西功能农产品检验检测中心, 山西农业大学, 山西 太原 030031; Shanxi Center for Testing of Functional Agro-Products, Shanxi Agricultural University, Taiyuan 030031, China; 山西功能农产品检验检测中心, 山西农业大学, 山西 太原 030031; Shanxi Center for Testing of Functional Agro-Products, Shanxi Agricultural University, Taiyuan 030031, China

**Keywords:** 手性固定相, 超高效液相色谱-串联质谱仪, 腈菌唑, 对映体, 小麦, chiral stationary phase, ultra-performance liquid chromatography-tandem mass spectrometry (UPLC-MS/MS), myclobutanil, enantiomers, wheat

## Abstract

建立了手性超高效液相色谱-串联质谱检测小麦及其加工制品中腈菌唑对映体残留的分析方法。样品经乙腈提取,*N*-丙基乙二胺(PSA)和C18净化,手性色谱柱Lux Cellulose-1(150 mm×2.0 mm, 3 μm)分离,质谱电喷雾正离子扫描(ESI^+^),多反应监测(MRM)模式进行检测。为准确定量,考察了小麦籽粒及其加工制品的基质效应,最终采用基质匹配校准法来补偿基质效应,并进行样品中腈菌唑对映体残留的校准定量。结果表明,腈菌唑的两个对映体得到良好的拆分。*S*-(+)-腈菌唑先出峰,*R*-(-)-腈菌唑后出峰,保留时间分别为4.34 min和5.13 min。*S*-(+)-腈菌唑和*R*-(-)-腈菌唑在小麦及其加工制品中的检出限均为0.2 μg/kg,定量限均为0.5 μg/kg。在0.5~25 μg/L范围内,腈菌唑对映体的峰面积与其质量浓度呈现良好的线性关系,相关系数(*R*^2^)均大于0.99。在对映体添加水平为5、50、100 μg/kg时,在小麦籽粒、麸皮、面粉、面团、馒头、面条、煮面水中,*S*-(+)-腈菌唑的平均回收率在82%~110%之间,RSD在0.9%~6.8%之间;*R*-(-)-腈菌唑的平均回收率在80%~109%之间,RSD在0.9%~6.8%之间。将该方法应用于实际样品的检测,在5份面粉、2份面条及2份馒头样品中均未检出腈菌唑对映体。该方法为手性农药腈菌唑对映体在初级农产品及其加工制品中的残留分析提供了有效的方法。

## Abstract (en)

A valid method based on ultra-performance liquid chromatography-tandem mass spectrometry (UPLC-MS/MS) with a chiral stationary phase was established for the determination of myclobutanil enantiomer residue in wheat grain and its processed products (flour, bran, pasta, steamed bun, noodle, and cooking water). The wheat grain and processed product samples were extracted with acetonitrile and purified with primary secondary amine (PSA) and C18. The enantiomers of myclobutanil were separated by Chiral column Lux Cellulose-1 (150 mm×2.0 mm, 3 μm, Phenomenex). The column temperature, sample volume injected, and flow rate were 30 ℃, 5 μL, and 0.25 mL/min, respectively. The mobile phase consisted of phase A (25%), water with 0.1% formic acid and 4 mM ammonium acetate, and phase B (75%), methanol with 0.1% formic acid and 4 mM ammonium acetate. A Waters Xevo TQ-S Micro MS/MS system (Waters, USA) was used for mass spectrometric analysis. An electrospray ionization (ESI) source operating in the positive ionization mode. MS analyses were performed in the multiple reaction monitoring (MRM) mode. The qualitative ions of myclobutanil were *m/z* 288.9/69.9 and 288.9/124.9, and the quantitative ion of myclobutanil was *m/z* 288.9/69.9. The source voltage was 3000 V, and the desolvation temperature was 400 ℃. The desolvation gas flow was 800 L/h, and the source temperature was 150 ℃. The matrix effect of wheat grains and their processed products on the determination of myclobutanil enantiomers by UPLC-MS/MS was investigated. *S*-(+)-myclobutanil and *R*-(-)-myclobutanil had a mid signal suppression effect on wheat grain, bran, pasta, steamed bun, and noodle, while *S*-(+)-myclobutanil and *R*-(-)-myclobutanil had a mid signal enhancement effect on flour and cooking water. Finally, the matrix-matched calibration method was effective in all matrices and was selected for the quantification of the myclobutanil enantiomer residue in the samples. The results showed that the two enantiomers of myclobutanil were well separated by this method. The first and second eluted enantiomers were *S*-(+)-myclobutanil and *R*-(-)-myclobutanil, respectively, with the corresponding retention times being 4.34 min and 5.13 min. The limits of detection (LOD) and limits of quantification (LOQ) of *S*-(+)-myclobutanil and *R*-(-)-myclobutanil in wheat and its processed products were 0.2 μg/kg and 0.5 μg/kg, respectively. In the linear range of 0.5-25 μg/L, the peak areas of the myclobutanil enantiomers showed a good linear relationship with the concentration, and the *R*^2^ values were all greater than 0.99. At fortification levels of 5, 50, and 100 μg/kg (enantiomer concentration), the average recoveries of *S*-(+)-myclobutanil in wheat grain and its processed products ranged from 82% to 110%, with RSDs between 0.9% and 6.8%. The average recoveries of *R*-(-)-myclobutanil in wheat grain and its processed products ranged from 80% to 109%, with RSDs between 0.9% and 6.8%. This method fulfils the requirements for pesticide residue analysis. The established method was applied to analyze five flour samples, two noodle samples, and two steamed bread samples. The results showed that *S*-(+)-myclobutanil and *R*-(-)-myclobutanil enantiomers were not detected in the samples. In this study, methods for the enantiomeric separation and residue analysis of myclobutanil in wheat were evaluated at the enantiomeric level, which enriched the methods of enantiomeric separation and residue analysis of chiral pesticide myclobutanil enantiomers in raw agricultural product (wheat grain) and its processed foods. This method is effective for the residue analysis of chiral pesticide myclobutanil enantiomers in raw agricultural commodities and its processed products.

手性是指实物与镜像对称却不能重叠的性质,手性分子存在一对基团组成相同但空间结构互为镜像关系的对映异构体,简称对映体^[1]^。具有这种性质的农药称为手性农药。手性农药在农药市场中占据重要地位,目前使用的农药中≥30%为手性农药^[2]^。手性农药对映体在非手性环境下的理化性质没有明显差异,但在手性条件下,尤其是生物体内酶、激素等手性环境中,其代谢降解、生物活性及毒性毒理等方面存在显著差别^[3]^。近年来,随着人们对手性化合物的深入了解,建立快速简单的手性农药对映体分离及残留分析方法越来越受到重视。常见的手性化合物拆分方法以色谱拆分法为主。色谱拆分法包括气相色谱法、高效液相色谱法、超临界流体色谱法、毛细管电泳法等,其中高效液相色谱法是应用最广泛的方法。目前高效液相色谱手性拆分方法中,手性固定相法由于具有简单、快速等优点被广泛使用^[4,5]^。

腈菌唑(myclobutanil)属于三唑类手性杀菌剂,分子中有1个手性中心,存在2个对映异构体*S*-(+)-腈菌唑和*R*-(-)-腈菌唑^[6]^,其结构式见图1。腈菌唑目前主要以外消旋体形式销售并使用,其登记作物主要有黄瓜、香蕉、小麦、梨等^[7]^。由于用量较小、药效较高、低毒、对小麦白粉病有较好的防治效果等优点,腈菌唑在小麦上应用广泛。研究表明,腈菌唑对映体在生物活性、毒性、降解等方面具有选择性:*S*-(+)-腈菌唑的杀菌活性明显优于*R*-(-)-腈菌唑^[8]^; *S*-(+)-腈菌唑对栅藻的毒性比*R*-(-)-腈菌唑高^[9]^; *S*-(+)-腈菌唑和*R*-(-)-腈菌唑在人肝微粒体中代谢具有对映选择性的差异,人类CYP450酶不代谢*R*-(-)-腈菌唑^[10]^; *R*-(-)-腈菌唑在蜥蜴中的富集高于其异构体^[11]^;在葡萄、大鼠肝细胞及肝微粒体中*S*-(+)-腈菌唑优先降解,*R*-(-)-腈菌唑相对富集^[6,12]^。为进一步深入研究手性农药腈菌唑对映体在残留降解、环境行为等方面的差异,建立其在不同基质中的分离及残留分析方法尤为重要。

**图 1:**
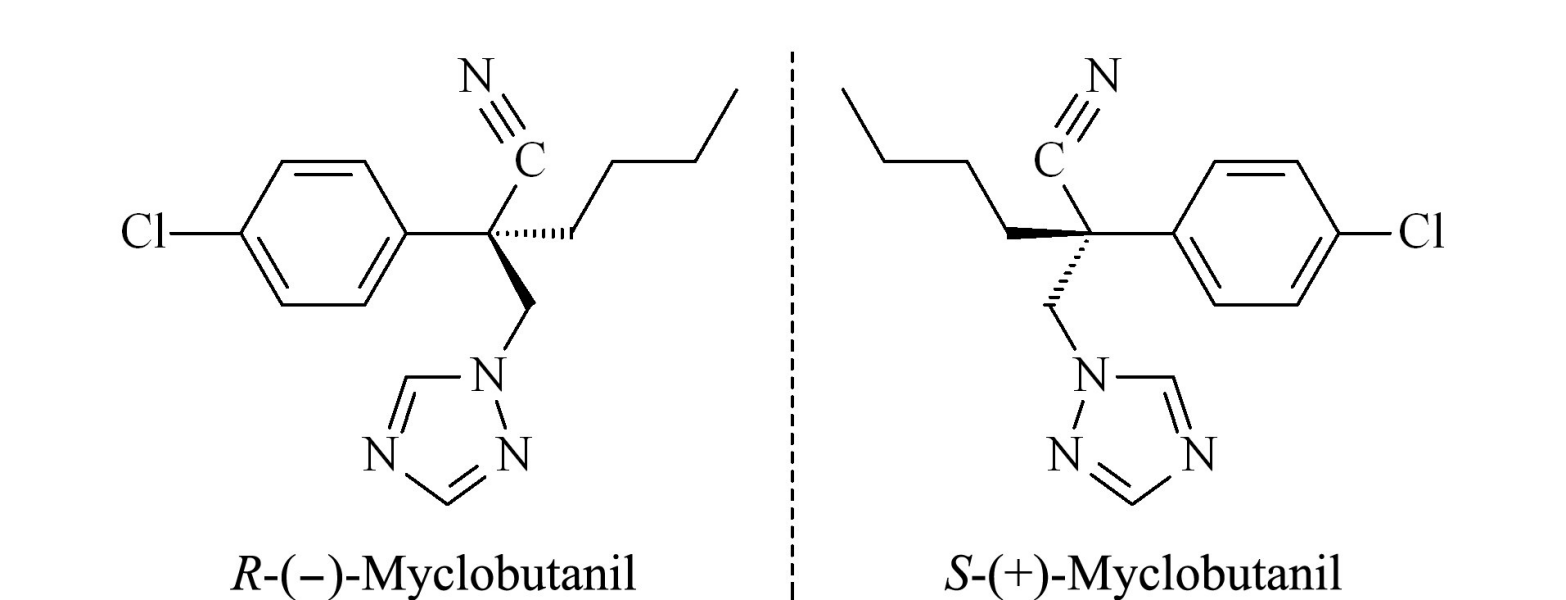
腈菌唑对映体的结构式

目前,手性农药腈菌唑对映体在不同基质中的手性残留分析已有报道。张文华等^[13]^利用超高效合相色谱(ACQUITYUPC^2^ Trefoil AMY1色谱柱)建立了腈菌唑对映体在黄瓜中的残留分析方法。张新忠等^[14]^采用超高效合相色谱(ChromegaChrial CCA色谱柱)-四极杆飞行时间质谱,建立了腈菌唑对映体在苹果、葡萄和茶叶中的分离及残留分析方法。Yang等^[15]^利用超高效合相色谱(ACQUITYUPC^2^ Trefoil AMY1色谱柱)-串联质谱建立了腈菌唑对映体在烟草中的检测方法。孙彦等^[16]^利用超高效液相色谱(Lux Cellulose-4手性色谱柱)-串联质谱建立了番茄、苦瓜、黄瓜和胡萝卜中腈菌唑对映体的分离方法。Li等^[17]^利用一种新型手性固定相(3,5-二氯苯氨基化纤维素键合硅胶固定相),用超高效液相色谱-串联质谱建立了腈菌唑对映体在10种蔬菜及水果中的手性分析方法。

本研究以小麦及其加工制品为研究对象,基于手性固定相,利用的UPLC-MS/MS建立了腈菌唑对映体的分离及残留分析方法,可用于不同初级农产品及加工食品中的手性农药对映体的检测。

## 1 实验部分

### 1.1 仪器、试剂与材料

Xevo TQ-S micro超高效液相色谱-串联三重四极杆质谱仪(UPLC-MS/MS,美国Waters公司);天平BS210S(精确到0.0001 g)及CAP8201(精确到0.1 g)(赛多利斯科学仪器北京有限公司);离心机5804 R(德国Eppendorf公司);多管涡旋混合仪(美国特朗纳有限责任公司); 10~100 μL及100~1000 μL移液器(德国Eppendorf公司)等。

腈菌唑标准品(99.9%,德国Dr. Ehrenstorfer GmbH);乙腈(HPLC级,美国Tedia公司);甲醇(HPLC级,赛默飞世尔科技(中国)有限公司)、甲酸(HPLC级,赛默飞世尔科技(中国)有限公司);乙酸铵(HPLC级,天津市光复精细化工研究所);氯化钠(分析纯,北京化工厂);乙二胺-*N*-丙基硅烷(PSA)和C18(分析纯,岛津技迩(上海)商贸有限公司)吸附剂;纯净水(广州屈臣氏食品饮料有限公司)。

供试小麦样品来自山西省农业科学院东阳试验基地,样品经脱粒后冷冻保存。

### 1.2 样品的制备

小麦籽粒样品:取部分小麦籽粒样品用粉碎机打碎,得到均质的小麦籽粒样品。

麸皮和面粉样品:部分小麦籽粒样品用磨粉机磨粉、过筛,得到麸皮和面粉样品。

馒头样品:称取100 g面粉,加入含有1 g干酵母的温水(约38 ℃)约48 mL,用筷子混合成面团后,手工揉3 min,于38 ℃恒温箱中醒发1 h,取出再揉3 min成型,在室温放置15 min,放入已煮沸并垫有纱布的蒸锅屉上蒸20 min (冒气时开始计时),取出并盖上干纱布冷却40~60 min。将馒头样品切成小碎块,备用。

面团样品:取300 g面粉,加入温水(约30 ℃),用筷子混合成面团后,手工揉10 min,在室温下静置20 min。将面团样品切成小碎块,备用。

煮面水及面条样品:取部分上述面团样品用小型面条机压片,将面片逐渐压薄至1.0 mm,在将面片压成2.0 mm宽的细长面条束。量取500 mL自来水于锅中,在电磁炉上煮沸,称取50 g面条样品,放入锅内,煮至面条芯的白色生粉刚刚消失,立即将面条捞出,以流动的自来水冲淋约10 s。得到煮面水及面条样品。再将面条样品切成小碎块,备用。

### 1.3 样品前处理

1.3.1 样品提取

小麦及其加工制品中腈菌唑对映体的提取方法采取改进的QuEChERS方法进行。

称取5 g样品(小麦籽粒、麸皮、面粉、馒头及煮面水),加入5 mL水(煮面水样品不加),加入10 mL乙腈,2500 r/min下涡旋5 min, 8000 r/min下离心5 min。

称取5 g样品(面团及面条),加入5 mL水,加入10 mL乙腈,12000 r/min下均质提取1 min,加入5 g氯化钠,2500 r/min下涡旋2 min, 8000 r/min下离心5 min。

1.3.2 样品净化

吸取上述提取液1 mL于盛有25 mg C18及25 mg PSA吸附剂的小离心管中,2500 r/min下涡旋2 min, 2500 r/min下离心2 min。吸取0.5 mL上清液,加入0.5 mL纯净水,混匀,过有机微孔滤膜(0.22 μm),待进UPLC-MS/MS分析。

### 1.4 仪器条件

色谱条件:Lux Cellulose-1手性色谱柱(150 mm×2.0 mm, 3 μm);流速为0.25 mL/min;进样量为5 μL;柱温30 ℃;流动相A为4 mmol/L的乙酸铵水溶液,B为4 mmol/L的乙酸铵甲醇溶液,均含体积分数为0.1%的甲酸;其比例为A: 25%, B: 75%。

质谱条件:电喷雾正离子扫描(ESI^+^);多反应监测(MRM)模式;毛细管电压3.0 kV;离子源温度400 ℃,脱溶剂气流量800 L/h;碰撞气为氩气,纯度>99.999%。腈菌唑定性离子对为*m/z* 288.9/124.9和*m/z* 288.9/69.6;锥孔电压均为36 V;碰撞能量分别为24 eV和38 eV;其中*m/z* 288.9/69.6为定量离子对。

### 1.5 标准溶液的配制

准确称取10 mg(精确到0.1 mg)腈菌唑标准品,用乙腈溶解并定容于10 mL容量瓶中,配成1 g/L的标准储备溶液,于≤-18 ℃条件下避光保存,备用。

## 2 结果与讨论

### 2.1 手性固定相的选择

利用色谱法可以有效地实现手性农药的分离与检测,而手性固定相是手性色谱分离的关键所在^[18]^。多糖类物质是液相色谱法中最常用的一类手性选择剂。Lux Cellulose-1色谱柱以手性选择剂纤维素-三(3,5-二甲基苯基氨基甲酸酯)修饰的硅胶为填料,是改良的多糖型手性固定相,在手性农药对映体的分离分析中具有较好的拆分效果^[19,20,21,22]^。故本研究选择Lux Cellulose-1作为手性分离色谱柱。

### 2.2 腈菌唑对映体的拆分

本研究利用手性色谱柱Lux Cellulose-1在甲醇/水体系下对腈菌唑对映体进行了拆分。已有文献^[20]^表明腈菌唑对映体在Lux Cellulose-1手性色谱柱上的出峰顺序不随流动相及柱温的改变而改变。由此可知,在本实验仪器条件下,*S*-(+)-腈菌唑对映体先出峰,*R*-(-)-腈菌唑对映体后出峰,保留时间分别为4.34 min和5.13 min。如图2所示,腈菌唑的两个对映体得到良好的拆分。

**图 2:**
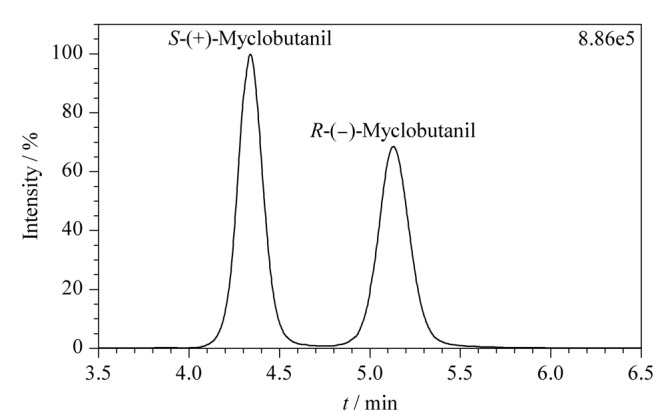
腈菌唑对映体的总离子流色谱图

### 2.3 基质效应

在UPLC-MS/MS分析中,尤其是使用ESI源时,由于共流出化合物在色谱系统中的离子竞争,基质效应(离子抑制或离子增强)普遍存在^[23]^。一般通过比较待测物在样品及纯溶剂中的响应值来确定基质效应的存在^[24]^。基质效应由Matuszewki等^[25]^修正的公式来计算:基质效应=(*R*_样品_/*R*_溶剂_-1)×100%,其中*R*_样品_为样品空白基质提取液中腈菌唑对映体的峰响应,*R*_溶剂_为乙腈溶液中腈菌唑对映体的峰响应。基质效应为负值时,产生离子抑制效应;基质效应为正值时,产生离子增强效应。基质效应在-20%~0或0~20%时为弱基质效应,在-50%~-20%或20%~50%时为中等基质效应,小于-50%或大于50%时为强基质效应^[26]^。

腈菌唑对映体在小麦籽粒及其相关加工制品中的基质效应见表1。腈菌唑对映体在小麦及其加工制品中的基质效应均在-20%~0或0~20%之间,表明存在弱基质效应。其中,*S*-(+)-腈菌唑和*R*-(-)-腈菌唑在小麦籽粒(wheat grain)、麸皮(bran)、面团(pasta)、馒头(steamed bun)和面条(noodle)中存在弱基质减弱效应,*S*-(+)-腈菌唑和*R*-(-)-腈菌唑在面粉(flour)和煮面水(cooking water)中存在弱基质增强效应。

**表 1:** 腈菌唑对映体的基质效应

Matrix	Matrix effects/%	
	S-(+)-Myclobutanil	R-(-)-Myclobutanil
Wheat grain	-7.7	-8.5
Flour	3.1	3.7
Bran	-4.1	-5
Pasta	-12.8	-13.4
Steamed bun	-7.1	-6.5
Noodle	-9.9	-12.1
Cooking water	1.6	2.6

基质效应产生的机理尚不明确且影响因素众多。为提高定量结果的准确度及精密度,在农药残留分析中通常采用基质匹配标准溶液校准方法来消除与补偿基质效应。因此,本文使用基质匹配标准溶液校准方法来定量分析物。

### 2.4 方法验证

分别用小麦籽粒、麸皮、面粉、面团、馒头、面条、煮面水空白样品基质提取液配制成腈菌唑对映体质量浓度为0.5、1.25、5、12.5、25 μg/L的标准工作溶液,以进样质量浓度(μg/L)为横坐标、化合物峰面积响应值为纵坐标绘制标准曲线。

在0.5~25 μg/L的范围内,腈菌唑对映体的峰面积与其质量浓度间呈现良好的线性关系,相关系数在0.9995~1之间(见表2)。

**表 2:** 腈菌唑对映体的线性范围、回归方程和相关系数(*R*^2^)

Matrix	Compound	Linear range/(μg/L)	Regression equation	R^2^
Wheat grain	S-(+)-myclobutanil	0.5-25	y=9.61×10^6^x+1.33×10^3^	0.9998
	R-(-)-myclobutanil	0.5-25	y=9.01×10^6^x+1.34×10^3^	0.9997
Flour	S-(+)-myclobutanil	0.5-25	y=9.99×10^6^x+4.09×10^2^	1.0000
	R-(-)-myclobutanil	0.5-25	y=8.83×10^6^x+4.33×10^2^	1.0000
Bran	S-(+)-myclobutanil	0.5-25	y=8.50×10^6^x+2.99×10^3^	0.9996
	R-(-)-myclobutanil	0.5-25	y=7.41×10^6^x+2.36×10^3^	0.9995
Pasta	S-(+)-myclobutanil	0.5-25	y=8.53×10^6^x+1.57×10^2^	1.0000
	R-(-)-myclobutanil	0.5-25	y=7.55×10^6^x-2.23×10^2^	1.0000
Steamed bun	S-(+)-myclobutanil	0.5-25	y=9.06×10^6^x+4.00×10^2^	1.0000
	R-(-)-myclobutanil	0.5-25	y=8.12×10^6^x+1.50×10^2^	0.9999
Noodle	S-(+)-myclobutanil	0.5-25	y=9.18×10^6^x-8.72×10^2^	0.9999
	R-(-)-myclobutanil	0.5-25	y=7.85×10^6^x-5.36×10^2^	1.0000
Cooking water	S-(+)-myclobutanil	0.5-25	y=1.02×10^7^x-6.25×10^2^	0.9998
	R-(-)-myclobutanil	0.5-25	y=9.08×10^6^x-7.38×10^2^	0.9998

*y*: peak area, *x*: mass concentration, μg/L.

以3倍信噪比及10倍信噪比确定方法的检出限和定量限。*S*-(+)-腈菌唑和*R*-(-)-腈菌唑在小麦及其加工制品样品中的检出限均为0.2 μg/kg,定量限均为0.5 μg/kg。

在小麦籽粒、麸皮、面粉、面团、馒头、面条、煮面水空白样本中添加腈菌唑标准溶液(添加水平为5、50、100 μg/kg),进行回收率测定,添加回收率结果见表3。*S*-(+)-腈菌唑在小麦籽粒及其加工制品中平均回收率在82%~110%之间,RSD在0.9%~6.8%之间;*R*-(-)-腈菌唑在小麦籽粒及其加工制品中平均回收率在80%~109%之间,RSD在0.9%~6.8%之间。结果表明该方法具有较好的准确性及重现性。

**表 3:** 腈菌唑对映体在小麦及其加工制品中的平均添加回收率(*n*=5)

Matrix	Spiked/ (μg/kg)	S-(+)-Myclobutanil			R-(-)-Myclobutanil	
		Average recovery/%	RSD/%		Average recovery/%	RSD/%
Wheat	5	90	5.2		90	4.5
grain	50	90	6.6		89	6.8
	100	88	3.7		86	3.4
Flour	5	98	4.3		101	5.0
	50	103	1.9		101	2.0
	100	98	0.9		97	0.9
Bran	5	91	6.8		92	5.4
	50	82	2.0		80	1.7
	100	84	1.7		82	1.4
Pasta	5	104	2.3		101	3.0
	50	106	2.4		104	2.5
	100	107	2.8		105	3.0
Steamed	5	104	4.1		104	4.4
bun	50	110	2.5		109	2.9
	100	109	2.3		107	1.9
Noodle	5	106	4.8		109	4.6
	50	108	4.6		108	4.4
	100	104	5.7		106	4.8
Cooking	5	90	4.4		90	4.5
water	50	99	5.5		97	5.5
	100	96	5.0		95	4.7

### 2.5 实际样品的检测

将该方法用于实际样品的检测。结果表明,在5份面粉、2份面条及2份馒头样品中均未检出(≤LOD)腈菌唑对映体。

## 3 结论

本文建立了基于手性固定相的UPLC-MS/MS测定小麦籽粒及其加工制品中腈菌唑对映体的残留分析方法。该方法高效、简单,同时具有较高的灵敏度及精密度,为手性农药腈菌唑对映体在初级农产品及其加工制品中的残留分析提供了有效的方法。
